# Prussian blue-supported platinum nanoparticles as pH-Universal catalase mimics: enabling robust chemiluminescent immunoassay for VEGF in clinical diagnostics

**DOI:** 10.3389/fbioe.2026.1762884

**Published:** 2026-03-04

**Authors:** Ying Yang, Jiyixuan Li, Xiang Hu, Yutao Xiu, Miao Zhang, Chao Wang, Xinlin Liu, Bing Liang, Dongming Xing, Tingting Zhang

**Affiliations:** 1 School of Basic Medicine, The Affiliated Hospital of Qingdao University, Qingdao University, Qingdao, China; 2 Molecular Medicine Innovation Center, Cancer Institute of The Affiliated Hospital of Qingdao University, Qingdao University, Qingdao, China; 3 School of Life Sciences, Tsinghua University, Beijing, China

**Keywords:** catalase mimics, chemiluminescent immunosensor, prussian blue, pt nanoparticle, vascular endothelial growth factor

## Abstract

**Introduction:**

Nanozymes have emerged as promising substitutes for natural enzymes in chemiluminescent immunoassays, offering distinct catalytic advantages and superior stability. Despite their potential, many conventional nanozymes are constrained by a strong dependence on pH, which limits their effectiveness in certain assay environments. This highlights the need for nanozymes that maintain robust catalytic activity under alkaline conditions and are compatible with luminol-based detection systems.

**Methods:**

In this study, we synthesized platinum nanoparticle-modified Prussian blue cubes (PB@Pt) and evaluated their enzyme-mimicking activity. The catalytic performance of PB@Pt was assessed under both weakly acidic and alkaline conditions. Its ability to enhance the luminol-H_2_O_2_ chemiluminescence (CL) system was investigated, and the CL signals were compared to those generated by natural horseradish peroxidase (HRP). Based on these properties, a novel CL immunoassay utilizing PB@Pt was developed for the sensitive detection of vascular endothelial growth factor (VEGF).

**Results and Discussion:**

The synthesized PB@Pt exhibited high catalase (CAT)-like activity across a broad pH range, including alkaline media. Remarkably, in alkaline conditions, PB@Pt catalyzed the luminol-H_2_O_2_ reaction, producing CL signals significantly stronger than those achieved with natural HRP. Leveraging this enhanced performance, the established PB@Pt-based CL immunoassay enabled a wide linear detection range, ultra-low detection limits, high specificity, and excellent stability for VEGF quantification. This work introduces a novel strategy for designing CAT-mimicking nanozyme probes, thereby broadening their utility in CL immunoassays and advancing the clinical translation of nanozyme-based diagnostics for applications such as biomarker screening and point-of-care testing (POCT).

## 1 Introduction

Chemiluminescence (CL) is a process in which luminescence is produced through a chemical reaction (Yang et al., 2020). It is widely used in clinical analysis due to its low background noise, high sensitivity, and broad dynamic range (Han et al., 2012). In CL systems, enzyme labeling is employed to amplify detection signals, which has become the mainstream strategy for chemiluminescent immunoassays (Passardi et al., 2005). Among these, the chemiluminescent sensing system relying on horseradish peroxidase (HRP) catalyzed luminol-H_2_O_2_ reactions is the most widely utilized (Zhang et al., 2018). It is well known that the optimal pH range for HRP-catalyzed reactions is between 6.0 and 6.5, whereas the luminol chemiluminescent reaction functions best at a pH of approximately 11 (He et al., 2013). These incompatible reaction conditions result in reduced luminescence efficiency in HRP-catalyzed luminol-H_2_O_2_ systems, ultimately lowering the sensitivity of this analytical method. This challenge limits the broader application of chemiluminescent immunoassays. Additionally, natural enzymes are often hindered by issues such as susceptibility to denaturation, poor stability, and high costs (Meng et al., 2011; Li et al., 2013; Harris et al., 2009).

The emergence of nanozymes offers a novel solution to these challenges. Nanozymes, a class of nanomaterials with enzyme-like activity, have been increasingly recognized as promising alternatives to natural enzymes in chemiluminescent immunoassays (Zhu et al., 2019). Nanozymes offer advantages including high catalytic activity, excellent stability, and low cost (Meng et al., 2023; Zha et al., 2022; Zhang et al., 2022). Typically, nanozymes demonstrate oxidase (OXD)-like and peroxidase (POD)-like activities under acidic conditions, whereas they exhibit catalase (CAT)-like activity in neutral or alkaline environments (Feng et al., 2024; Zhang et al., 2023).

Notably, the CAT-mimicking activity of nanozymes in alkaline environments can generate reactive oxygen species (ROS) through catalyzing H_2_O_2_ decomposition, effectively driving signal amplification in chemiluminescence systems. This mechanism establishes a novel pathway for highly sensitive detection of biomarkers. A representative example is the AuPtCo nanopolyhedrons material developed by Bi’s research team (Zhu et al., 2019). This nanozyme demonstrates exceptional CAT-like activity at pH 13.0, with its catalytic process generating hydroxyl radicals (HO•), superoxide anions (O_2_^•-^), and singlet oxygen (^1^O_2_) that significantly enhance signal intensity in the ABEI-H_2_O_2_ chemiluminescence system, having been successfully applied for precise detection of H_2_O_2_ and lipoprotein-associated phospholipase A_2_ (Lp-PLA_2_). Given this versatility, developing nanozymes with CAT-like activity under alkaline conditions is essential for optimizing the luminol-H_2_O_2_ chemiluminescent sensing system.

Among the various nanozymes, platinum nanoparticles (PtNPs) stand out due to their remarkable versatility and high catalytic efficiency, positioning them as highly promising for applications in biosensing and nanomedicine (Wang Y. et al., 2021; Zhang et al., 2010; Moglianetti et al., 2016). Notably, their significant CAT-like activity under neutral and alkaline conditions is of particular interest (Li J. et al., 2015; Liu et al., 2014). Their small size minimizes platinum usage, lowering material costs, while ensuring a high density of active sites to maintain superior catalytic performance (Wu et al., 2023). However, the small size of PtNPs comes with the challenge of aggregation (Chen et al., 2023). The high surface energy of PtNPs drives them to aggregate, potentially causing structural deformation or transformation. This aggregation can severely reduce the number of exposed catalytic active sites, thereby reducing overall catalytic activity or even rendering the particles inactive. Immobilizing small PtNPs on stable substrates, such as metal-organic frameworks (MOFs), is an effective strategy to address this issue (Aijaz et al., 2012). This approach stabilizes PtNPs, preserving catalytic activity while preventing aggregation and structural changes. In this context, Prussian blue (PB) nanoparticles, known for their excellent stability and biocompatibility, present an attractive option as substrates for PtNPs.

PB exhibits several advantageous properties, including excellent biocompatibility, high chemical stability, and ease of preparation (Shen et al., 2022). Additionally, PB also demonstrates exhibits enzyme-mimicking activities, displaying CAT-like, superoxide dismutase (SOD)-like, and POD-like activities (Zhang et al., 2016). These properties make PB highly effective in addressing reactive oxygen species (ROS)-related diseases, which has been the primary focus on this area (Hou et al., 2022; Liu Q. et al., 2023; Hu et al., 2024). However, its potential application in chemiluminescent immunoassays, particularly through its CAT-like activity, remains largely unexplored. Therefore, synthesizing a PB@Pt nanocomposite to harness their combined catalytic effects in the luminol-H_2_O_2_ chemiluminescent sensing system represents a promising research direction.

In this study, we first synthesized PB nanocubes *via* a hydrothermal method, followed by *in situ* growth of PtNPs on their surface through a reduction process, successfully creating PB-supported PtNPs (PB@Pt) nanozyme. The porous structure of PB (Reddy et al., 2020) effectively increases the number of active sites, while its anisotropic cubic structure further amplifies this effect. Compared to spherical structures, cubic ones provide a larger specific surface area for the same volume (Wang et al., 2019), which not only stabilizes the nucleation and growth of PtNPs but also prevents their aggregation. The synthesized PB@Pt nanozyme exhibited excellent CAT-like activity under acidic, neutral, and alkaline conditions and could catalyze the chemiluminescence reaction in the luminol-H_2_O_2_ system, thus enabling the development of a chemiluminescent sensing platform. This platform provides the foundation for improving the sensitivity and specificity of biosensing applications, thus holds great potential for detecting and monitoring biomarkers and disease indicators. In recent years, the collaborative application of antibodies, nucleic acids and nanomaterials in the field of diagnosis is promoting the rapid development of detection technology in the direction of high sensitivity and reliability. Antibodies provide specific recognition, while nucleic acid probes incorporate enzyme-assisted signal amplification (e.g., exonuclease cycling strategies) (Wan et al., 2025) enabling ultra-sensitive detection of low-abundance targets (e.g., miRNAs, antibiotics) (Huang et al., 2025). With the help of the signal enhancement and anti-interference capability of nano materials, these methods significantly improve the performance and stability of the sensor, provide reliable tools for early disease diagnosis, food safety monitoring, etc., and show a wide prospect from laboratory to practical application.

To validate the feasibility of this approach, we conducted experiments using vascular endothelial growth factor (VEGF) as a model biomarker. VEGF is a signaling protein secreted by endothelial and tumor cells, playing a key regulatory role in angiogenesis and serving as an important biomarker for cancer diagnosis (Wang et al., 1998; Vikkula et al., 1996). VEGF is widely used in the detection of various human diseases, including cancer (Carmeliet and Jain, 2000; Plate et al., 1992; Salven et al., 1999), central nervous system disorders (Merrill and Oldfield, 2005), psoriasis (Detmar, 2004), and retinal diseases (Ray et al., 2004). Therefore, serum levels of VEGF are of significant diagnostic and therapeutic value in disease monitoring. Our proposed chemiluminescent sensing platform demonstrated exceptional sensitivity and selectivity in detecting VEGF, with a broad detection range (5–2,300 pg mL^-1^) and a detection limit as low as 5 pg mL^-1^. Utilizing the strong CAT-like activity of PB@Pt nanozyme under alkaline conditions, this method addresses the compatibility issues associated with traditional HRP and luminol in chemiluminescence reactions, significantly enhancing detection sensitivity and broadening the potential applications of nanozymes.

## 2 Experimental section

### 2.1 Synthesis of PB nanocubes

PB nanocubes were synthesized using a hydrothermal method, as described in the literature (Wang T. et al., 2021). 1.58 g of K_3_Fe(CN)_6_·3H_2_O and 36 g of PVP were dissolved in 480 mL of 0.01 M HCl and stirred at 550 rpm for 30 min. The mixture was then heated in an oil bath at 80 °C for 20 h. After the reaction, the solution was cooled to room temperature and the PB nanocubes were collected by centrifugation at 13,000 rpm for 15 min, with the process repeated three times. The final product was then dried at 60 °C overnight.

### 2.2 Synthesis of PB@Pt

**SCHEME 1:**
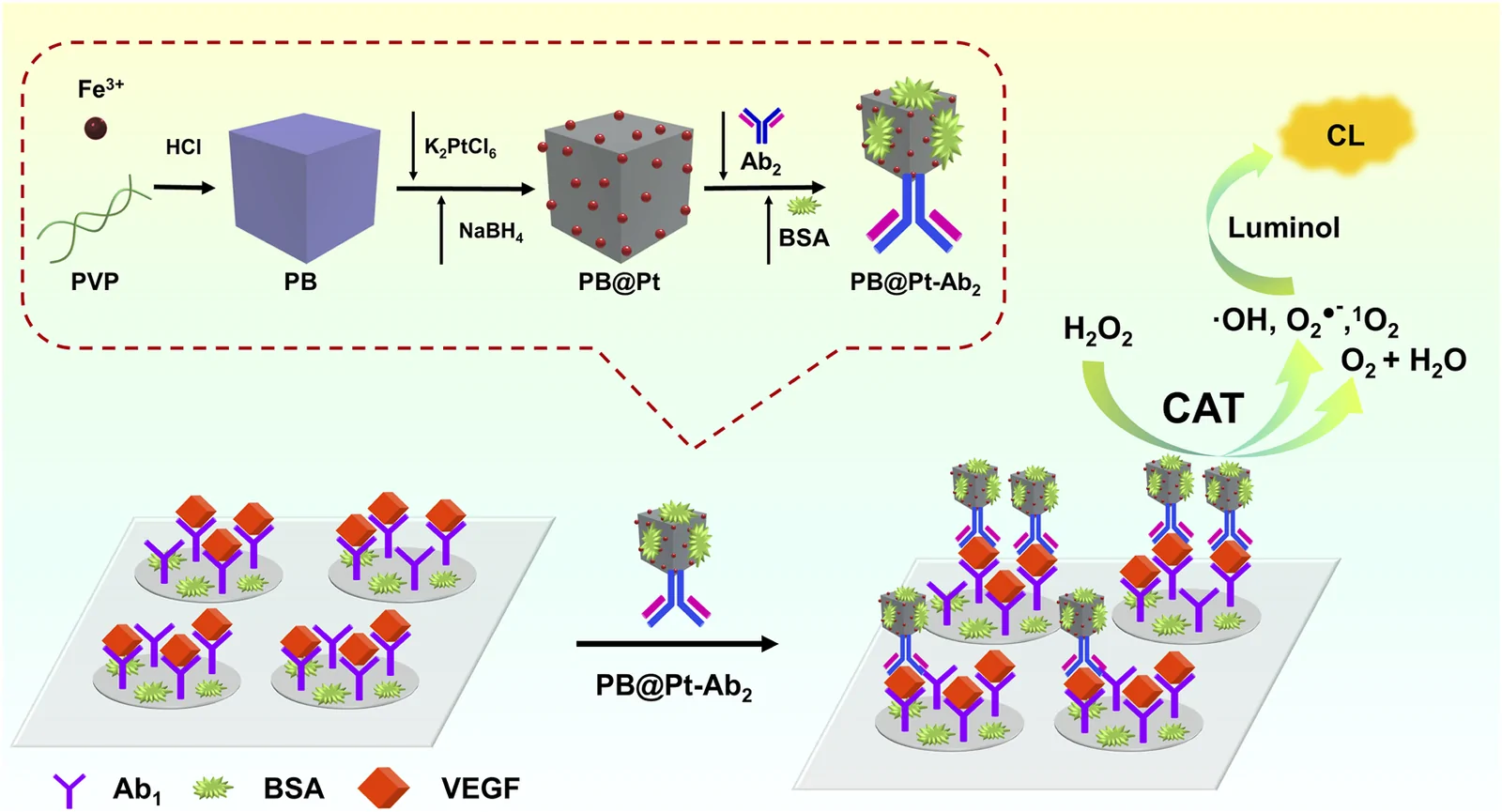
Schematic illustration of the Synthesis Process of PB@Pt and the CAT-like Activity of PB@Pt for Chemiluminescent Sensing of VEGF.

First, disperse 20 mg of PB in 20 mL of deionized water by ultrasonication for 5 min (Scheme 1). While stirring, combine the dispersion with 10 mL of an aqueous solution containing 50 mg K_2_PtCl_6_ and stir at 550 rpm for 1 h. Then, slowly add 2 mL of freshly prepared ice-cold NaBH_4_ solution (4 mg mL^-1^) to the mixture while stirring for another 30 min. After the reaction, wash the resulting products by centrifugation with deionized water at 13,000 rpm for 15 min, repeating the process three times. Finally, the product PB@Pt50 was dried at 60 °C overnight. To investigate the effect of Pt content on nanozyme activity, the amount of K_2_PtCl_6_ added during synthesis was adjusted to 30 mg, 40 mg, and 60 mg, yielding PB@Pt30, PB@Pt40, and PB@Pt60 nanomaterials. (Unless specified otherwise, PB@Pt refers to PB@Pt50).

### 2.3 CAT mimetic performance study of PB@Pt

The CAT mimetic performance of PB@Pt was evaluated by measuring the oxygen levels with a dissolved oxygen meter (JPSJ-606T, Shanghai Lei-Ci, China) at room temperature. A typical reaction mixture for the assay was prepared by combining 2.0 mL of PBS (0.1 M, pH 9.0), 25 mM H_2_O_2_ and 2.5 μg mL^-1^ PB@Pt was prepared and allowed to react for 12 min. The effect of Pt content on enzymatic activity was investigated under identical conditions. The influence of pH on the CAT-mimetic activity of PB@Pt was also examined by varying the pH while following the same procedure.

### 2.4 Preparation of nanozyme probe (PB@Pt-Ab_2_)

The PB@Pt-Ab_2_ nanozyme probe was prepared using a previously reported method with minor modifications (Shi et al., 2023). 40 µg of VEGF antibody (Ab_2_) was added to 1 mL of PB@Pt aqueous solution (1 mg mL^-1^). The mixture was stirred until fully dissolved and incubated at 37 °C for 1 h. Next, 100 µL of 10% BSA aqueous solution was added, and the mixture was incubated with shaking at room temperature for 1 h. The solution was centrifuged at 9,000 rpm for 15 min at 4 °C to remove unbound Ab_2_. The precipitate was resuspended in 400 µL of 0.01 M PBS (pH 7.4, containing 1% BSA) to obtain the PB@Pt-Ab_2_ nanozyme probe stock solution, stored at 4 °C. For subsequent use, the stock solution was diluted 1:200 with 0.01 M PBS (pH 7.4, containing 1% BSA and 0.01% Proclin-300) to prepare the final PB@Pt-Ab_2_ nanozyme probe solution, stored at 4 °C until needed.

### 2.5 Chemiluminescent detection of VEGF

Add 50 µL of VEGF standard solution or human serum samples to a 96-well plate coated with VEGF antibody (Ab_1_) (Scheme 1). Then, add 50 µL of PB@Pt-Ab_2_ nanozyme probe and mix thoroughly for 30 s. Seal the plate with film and incubate at 37 °C for 60 min to form a stable sandwich immunocomplex. After incubation, wash and dry the wells. Next, add 25 µL of 3.0 mM luminol (prepared in 0.1 mM NaOH, pH 10.0) and 25 µL of 7.5 mM H_2_O_2_ (prepared in 0.1 mM NaOH, pH 10.0) to initiate the chemiluminescence (CL) reaction. Finally, measure the chemiluminescence signal using a chemiluminescence detection instrument.

## 3 Results and discussion

### 3.1 Characterization of the PB@Pt

**FIGURE 1:**
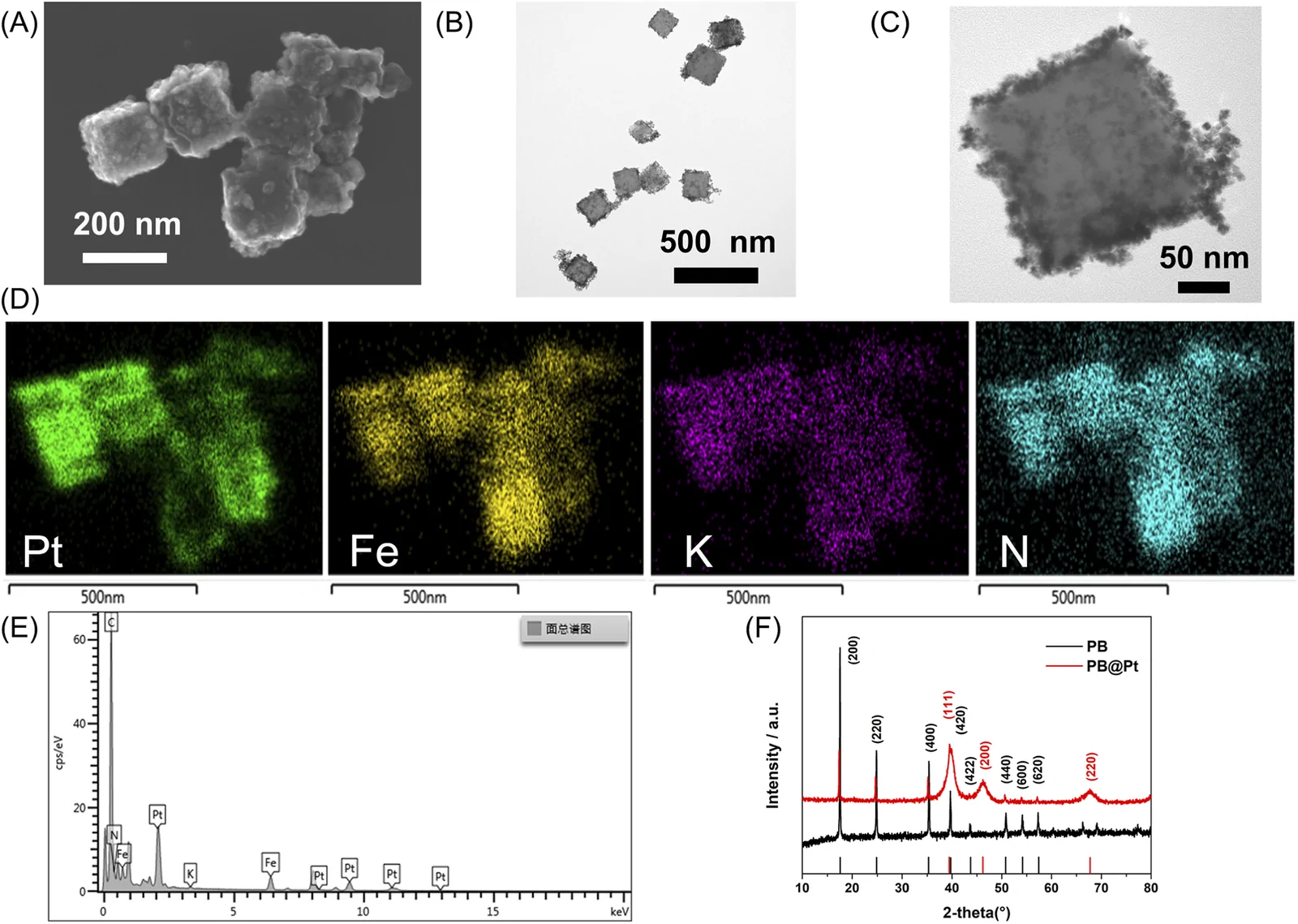
Characterization of PB@Pt. **(A)** SEM and **(B) (C)** TEM images of PB@Pt. **(D)** The EDX elemental maps and **(E)** EDX spectrum of PB@Pt. **(F)** XRD spectra of PB and PB@Pt.

The morphology, composition, and structure of PB@Pt were analyzed using SEM, TEM, EDX, and XRD. As shown in Figures 1A,B, PB@Pt retains the cubic structure of PB (Supplementary Figures S1A,B); however, the incorporation of PtNPs results in a rougher surface and an increase in particle size from 135 nm to 170 nm. Figure 1C reveals that the ultra-small PtNPs are uniformly distributed on the PB surface, without forming isolated nanoparticles, which is highly beneficial for the development of nanozyme probes. EDX mapping (Figure 1D) confirms the uniform distribution of Pt, Fe, K, and N elements, and the EDX spectrum (Figure 1E) verifies the presence of Pt, Fe, K, N, and C elements. Figure 1F presents the XRD patterns of PB and PB@Pt. The peaks at 17.4, 24.9, 35.3, 39.7, 43.6, 50.7, 54.0, and 57.3° correspond to the (200), (220), (400), (420), (422), (440), (600), and (620) planes, respectively (JCPDS card no. 73–0,687) (Bai et al., 2021). This pattern confirms the good crystallinity of PB. For PB@Pt, the primary peaks of PB are retained, indicating that the introduction of Pt does not significantly alter its crystallographic structure. Distinct peaks of crystalline metallic Pt are observed (JCPDS card no. 87–0,646) (Liu J. et al., 2023; Chen et al., 2021), corresponding to the (111), (200), and (220) planes at 39.5, 46.2, and 67.7°, respectively.

### 3.2 CAT mimetic activities of PB@Pt

**FIGURE 2:**
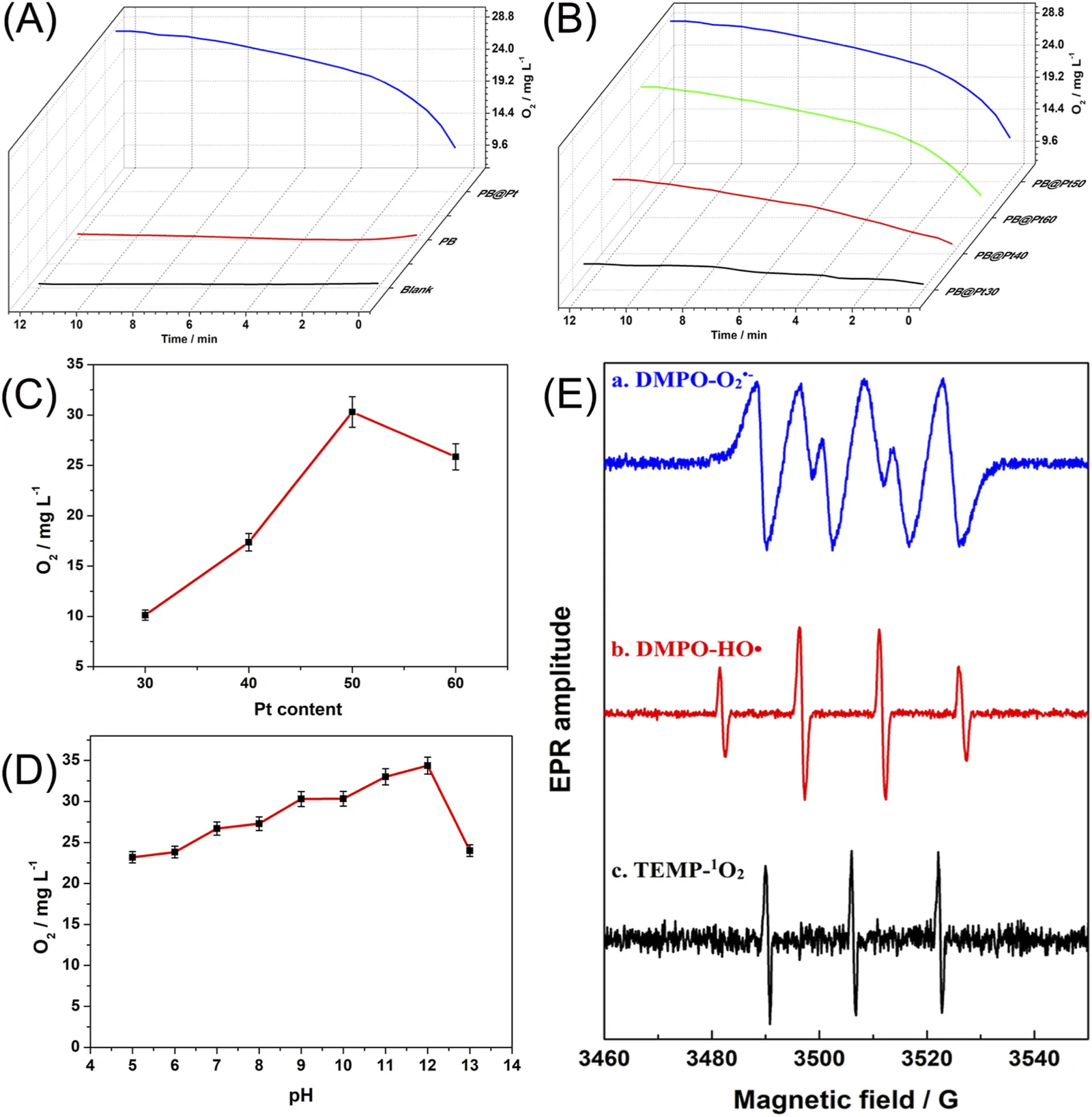
**(A)** CAT-mimetic properties of the PB and PB@Pt. **(B)** Effect of Pt content on the CAT-mimetic activity of PB@Pt and **(C)** oxygen content measured at 12 min. **(D)** Effect of pH on the CAT-mimetic activity of PB@Pt. **(E)** EPR spectra of different radical adducts: **(A)** DMPO-O_2_^•-^, **(B)** DMPO-HO•, and **(C)** TEMP-^1^O_2_ in the PB@Pt-catalyzed H_2_O_2_ reaction.

We investigated the CAT-like activity of PB@Pt. As shown in Figure 2A, PB@Pt catalyze the decomposition of H_2_O_2_ to generate O_2_, with O_2_ content gradually increasing over time, confirming its CAT-like activity. Compared to PB, the CAT-like activity of PB@Pt was significantly enhanced. Additionally, at higher concentrations of PB and H_2_O_2_, PB itself also demonstrated some CAT-like activity (Supplementary Figure S1C). Furthermore, we studied the effect of Pt content on the enzymatic activity of the material. As shown in Figures 2B,C, when 50 mg K_2_PtCl_6_ was added, the CAT-like activity of the resulting PB@Pt material reached its highest level. Therefore, PB@Pt50 was selected as the material for subsequent experiments and is referred to as PB@Pt for convenience in the text. In addition, we tested the effect of pH on the CAT-like activity of PB@Pt. Figure 2D shows that PB@Pt exhibited CAT-like activity under weakly acidic, neutral, and alkaline conditions, with the highest activity observed at pH 12.

Additionally, two spin-trapping agents, 5,5-dimethyl-1-pyrroline-N-oxide (DMPO) and 2,2,6,6-tetramethylpiperidine (TEMP), were utilized as probes to detect oxygen-related free radicals generated during the decomposition of H_2_O_2_ catalyzed by PB@Pt, using electron paramagnetic resonance (EPR) spectroscopy. DMPO was employed to capture short-lived, highly reactive superoxide anion radicals (O_2_^•-^) and hydroxyl radicals (HO•), resulting in the formation of DMPO-O_2_^•-^ and DMPO-HO• spin adducts. These adducts exhibit distinct EPR spectra characterized by hyperfine splitting patterns of 1:1:1:1 for DMPO-O_2_^•-^ and 1:2:2:1 for DMPO-HO•. Additionally, TEMP reacts with singlet oxygen (^1^O_2_), resulting in TEMP-^1^O_2_ adducts, that generate a characteristic 1:1:1 triplet signal. As shown in Figure 2E, the EPR spectra for DMPO-O_2_^•-^ (curve a), DMPO-HO• (curve b), and TEMP-^1^O_2_ (curve c) confirmed the presence of O_2_^•-^, HO•, and ^1^O_2_ radicals during the PB@Pt-catalyzed decomposition of H_2_O_2_, thus validating the generation of these oxygen-related free radicals in the process. Based on previous research, we hypothesize that PB@Pt could serve as a catalyst for chemiluminescence detection (Zhou et al., 2022).

### 3.3 Comparison of the catalytic effects of PB@Pt nanozyme and HRP on the luminol-H_2_O_2_ system

**FIGURE 3:**
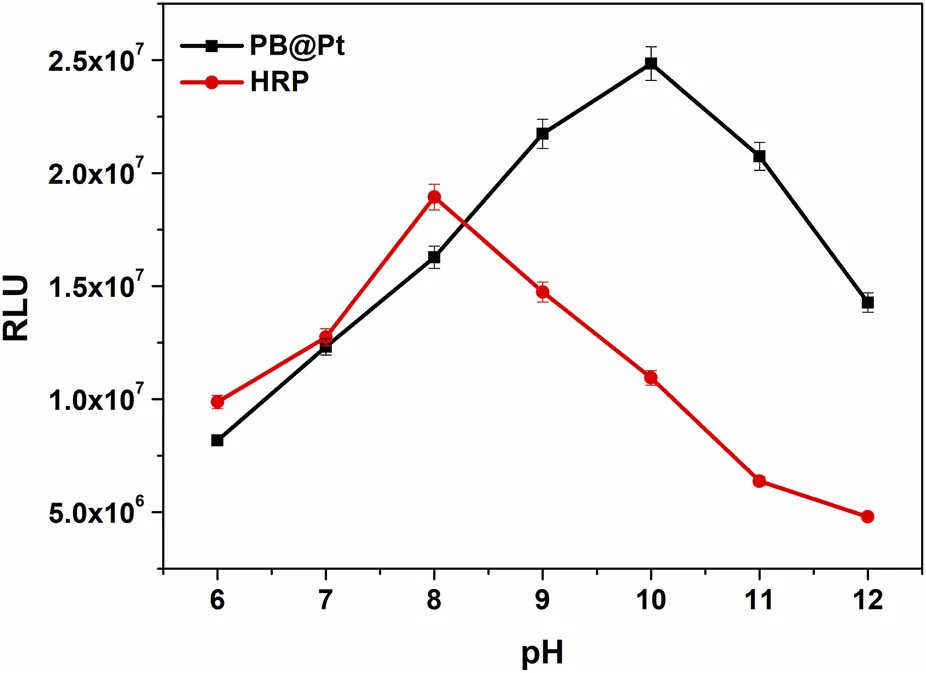
Effect of pH on the CL intensity of luminol-H_2_O_2_ system catalyzed by PB@Pt and HRP.

First, we verified the feasibility of using PB@Pt for chemiluminescence detection by comparing the catalytic effects of PB@Pt and HRP on the luminol-H_2_O_2_ system under different pH levels. As shown in Figure 3, the chemiluminescence intensity catalyzed by HRP was highest at pH 8, whereas PB@Pt demonstrated good catalytic activity across a broader pH range of 9–11, with its maximum activity occurring at pH 10. Compared to HRP, PB@Pt displayed higher catalytic activity in a pH range better suited to the luminol-H_2_O_2_ system, and its catalytic performance was significantly superior to that of HRP.

We compared the chemiluminescence intensity of Luminol-H_2_O_2_ system catalyzed by the same concentration of HRP and PB@Pt (as shown in Figure 3) at pH 6-12. The results showed that Luminol-H_2_O_2_ double substrate reached the optimal catalytic activity at pH 10 with the increase of pH (6–12) PB@Pt, and the optimal catalytic activity of HRP at pH 8. Because the optimal pH of Luminol-H_2_O_2_ is 10–11, and the optimal catalytic effect of HRP peroxidase is between 7-8, PB@Pt is more suitable for Luminol-H_2_O_2_ catalytic system, and the catalytic activity of PB@Pt is obviously higher than that of HRP at pH10.

### 3.4 Characterization of PB@Pt-Ab_2_ signal probe

**FIGURE 4:**
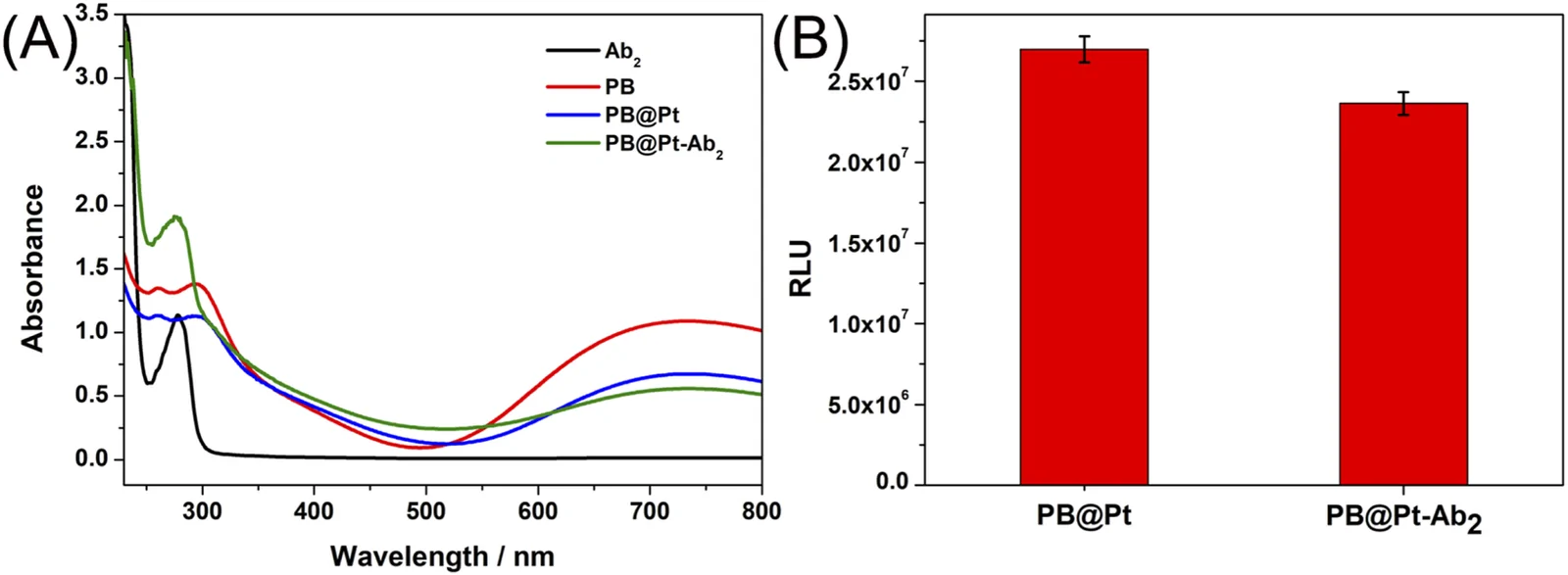
**(A)** UV−vis spectroscopy of different materials. **(B)** CL performance of PB@Pt and PB@Pt-Ab_2_.

We analyzed the preparation of the PB@Pt-Ab_2_ signal probe using UV-vis spectroscopy. As shown in Figure 4A, the Ab_2_ exhibits a distinct protein absorption peak at 278 nm (Shi et al., 2023). PB and PB@Pt exhibit characteristic absorption peaks at 260 nm, 294 nm, and 720 nm (Zhu et al., 2019; Wang et al., 2022; Pan et al., 2020). PB@Pt-Ab_2_ exhibits absorption peaks at both 278 nm and 720 nm, confirming the successful immobilization of Ab_2_ on PB@Pt. These results confirm the successful preparation of the PB@Pt-Ab_2_ signal probe. Furthermore, we compared the chemiluminescence intensity of PB@Pt and PB@Pt-Ab_2_. As shown in Figure 4B, after conjugating with the VEGF antibody (Ab_2_), PB@Pt-Ab_2_ retained a high level of chemiluminescence intensity, indicating that the nanozyme probe maintained robust CAT-like enzymatic activity.

It should be noted that binding of the detection antibody to the PB@Pt surface may affect its peroxidase-like activity and the accessibility of the active site through both steric hindrance and modification of the surface microenvironment. However, our experimental results show that this effect is manageable. Under the optimal experimental conditions, the complex retains most of the catalytic activity (about 87.6%), which lays the foundation for the subsequent efficient chemiluminescence immunoassay. The excellent sensitivity and detection limit of the final sensor demonstrated that PB@Pt-Ab2 can still effectively catalyze the luminol-H2O2 luminescence reaction, indicating that the active site maintains good accessibility.

### 3.5 Optimization of experimental conditions

**FIGURE 5:**
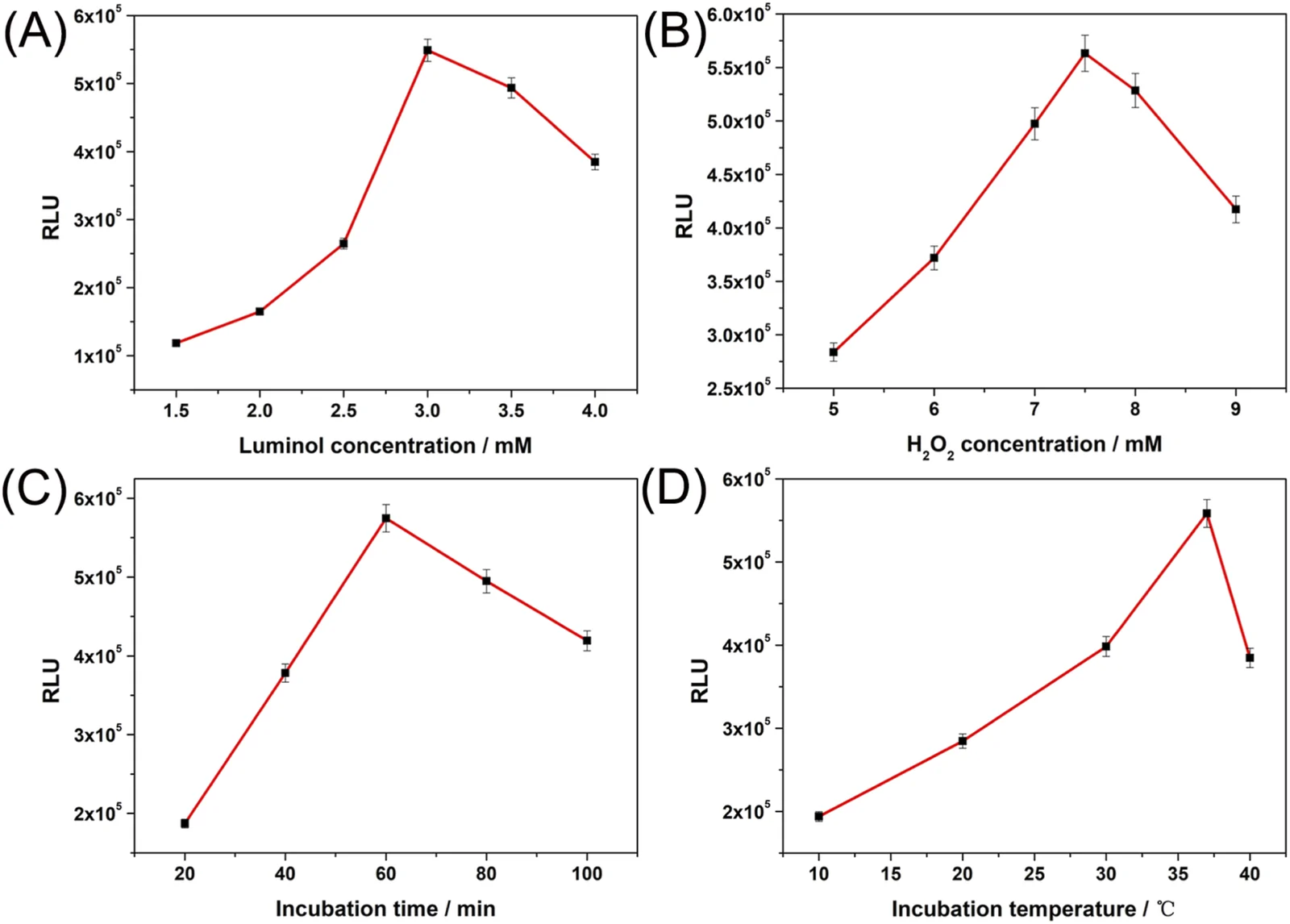
Effects of **(A)** luminol concentration, **(B)** H_2_O_2_ concentration, **(C)** incubation time, and **(D)** incubation temperature on the CL intensity of luminol-H_2_O_2_ system catalyzed by PB@Pt.

The key parameters influencing the performance of the chemiluminescent immunosensor were optimized. The concentration of luminol, a crucial chemiluminescent reagent, directly affected the sensor’s response. Experimental data revealed that the optimal luminol concentration was 3.0 mM (Figure 5A). Similarly, the concentration of H_2_O_2_, serving as the oxidant in the chemiluminescent reaction, significantly impacted the sensor’s signal, with the optimal concentration determined to be 7.5 mM (Figure 5B). We further investigated the effects of incubation time and temperature on the formation of the sandwich-type immunocomplex. The chemiluminescent intensity increased with longer incubation times, reaching a maximum at 60 min, which was therefore selected as the optimal incubation time for subsequent experiments (Figure 5C). Figure 5D shows that increasing the incubation temperature from 10 °C to 37 °C gradually enhanced the chemiluminescent signal, with the strongest signal observed at 37 °C. Beyond this point, the signal declined, establishing 37 °C as the optimal temperature for the chemiluminescent immunosensor.

### 3.6 Analytical performance of the chemiluminescence immunosensor

**FIGURE 6:**
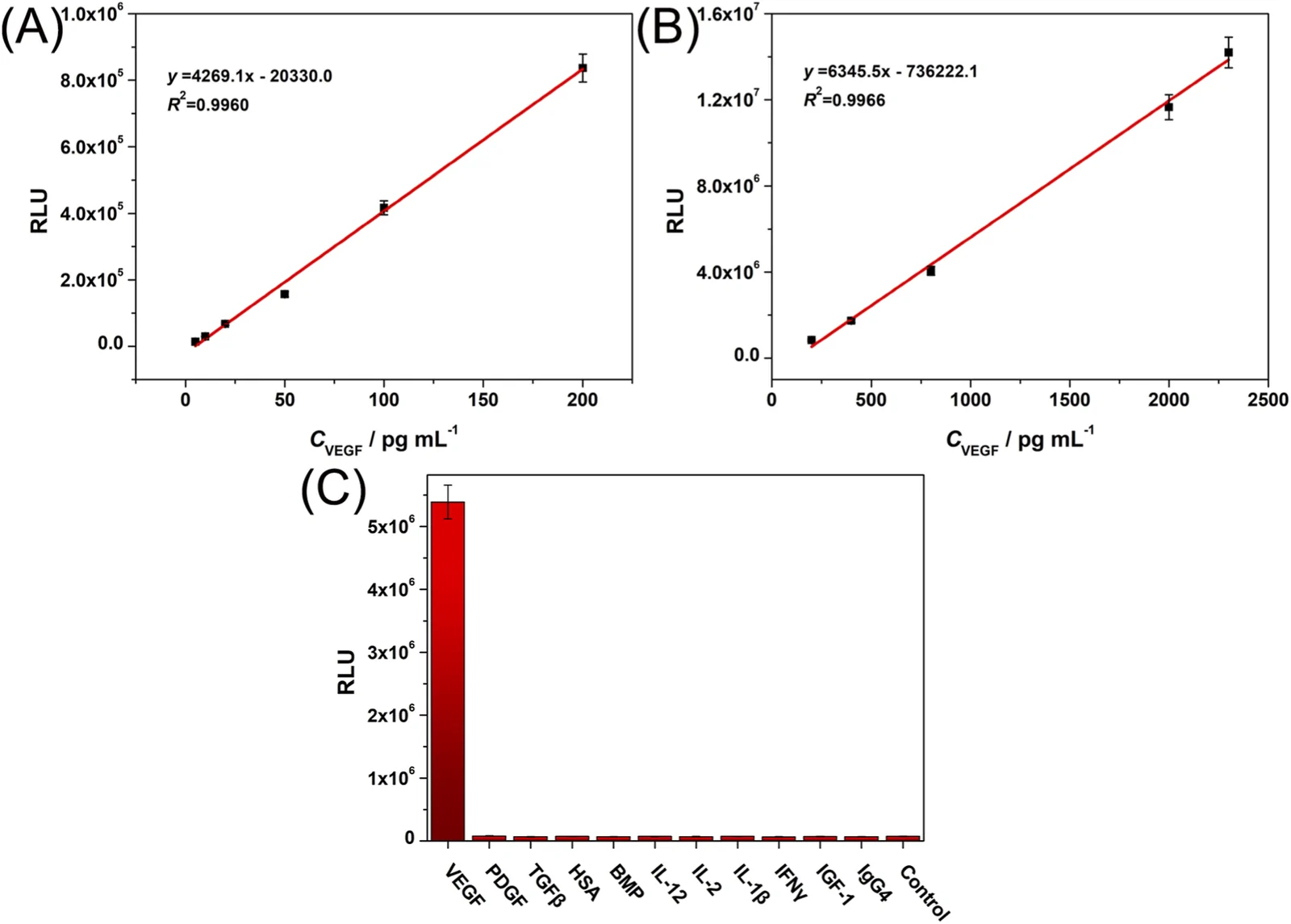
**(A,B)** The calibration curve of the proposed chemiluminescence immunosensor for VEGF detection. The concentrations of VEGF: 5, 10, 20, 50, 100, 200, 400, 800, 2000, 2,300 pg mL^-1^. **(C)** Selectivity test for VEGF detection.

Under optimal conditions, we developed a sandwich-type CL immunosensor for detecting VEGF, utilizing PB@Pt as a signal amplification catalyst in place of HRP. As demonstrated in Figures 6A,B, the CL intensity increased with rising VEGF concentrations. Within the range of 5–200 pg mL^-1^ and 200–2,300 pg mL^-1^, the CL intensity exhibited strong linear correlation with the VEGF concentration. The detection limit of this method was determined to be 5 pg mL^-1^ (S/N = 3). Compared to previously reported VEGF detection methods (see Supplementary Table S1), this approach provides both a lower detection limit and a broader linear range.

### 3.7 Specificity, reproducibility, and stability of the immunosensor

The specificity of the proposed CL immunosensor for VEGF was assessed by evaluating cross-reactivity between the target antigen and several non-specific antigens. As shown in Figure 6C, equivalent concentrations (1,000 pg mL^-1^) of platelet-derived growth factor (PDGF), transforming growth factor-β (TGF-β), human serum albumin (HSA), bone morphogenetic protein (BMP), interleukin-12 (IL-12), IL-2, IL-1β, interferon-γ (IFN-γ), insulin-like growth factor-1 (IGF-1), and immunoglobulin G4 (IgG4) were used as potential interferents, with PBS serving as a blank control. The results demonstrated that none of the interferents produced significant changes in CL signals compared to the blank, while VEGF generated a strong CL signal. This confirms that cross-reactivity between the capture antibody and non-specific antigens was negligible, validating the high specificity of the CL immunosensor for VEGF.

Furthermore, the reproducibility and stability of the CL immunosensor were evaluated (Supplementary Figure S2). The relative standard deviations (RSD) for measurements of 200 pg mL^-1^ of VEGF, taken within a single day and over a span of 5 days, were found to be 4.32% and 5.79%, respectively, demonstrating the satisfactory reproducibility and stability of the proposed immunosensor.

### 3.8 Detection of VEGF in serum samples

To further investigate the clinical application potential of the proposed CL immunosensor, VEGF concentrations in five human serum samples were measured and compared with those obtained using a commercial VEGF detection kit (Shandong Yida Biotechnology). The experimental results, presented in Supplementary Table S2 and Supplementary Figure S5, reveal no significant differences between the detection outcomes of the two methods. This finding further validates the feasibility and accuracy of the developed CL immunosensor for clinical applications.

## 4 Conclusion

In summary, we successfully prepared PB@Pt nanozyme with high CAT-like activity using hydrothermal and reduction methods. The porous cubic structure of PB not only provides an increased number of active sites but also stabilizes the nucleation and growth of PtNPs due to its larger specific surface area, effectively preventing their aggregation. The synergistic effect between PtNPs and PB imparts PB@Pt with robust CAT-like activity across weakly acidic, neutral, and alkaline conditions, demonstrating significantly higher catalytic activity for the luminol-H_2_O_2_ system compared to HRP under alkaline conditions. Leveraging the excellent properties of PB@Pt, we successfully constructed a CL biosensor for VEGF, achieving high sensitivity and selectivity in detecting VEGF in serum samples.

Compared with classical Pt nano-enzyme, TGA-CdTe quantum dot cross-linked catalase, VEGF165 specific binding aptamer (Apt) vascular endothelial growth factor (VEGF165) quantitative detection method and enzyme-linked chemiluminescence immunoassay (ELCLIA) (see Supplementary Table S1), our material has certain advantages in terms of working pH, lower detection limit and wide linear range. The optimal pH of many high-performance nano-enzymes (such as some carbon-based materials) is in the acidic range, and PB@Pt-Ab_2_ can perform optimally in the alkaline environment (pH 10–11) required for chemiluminescence immunoassay, which is more suitable for practical application.

The catalytic activity of PB@Pt is attributed to its unique core-shell synergetic effect. PB not only acts as a carrier for stabilizing Pt NPs, but its Fe^3+^/Fe^2+^ redox couple constitutes an efficient “electron shuttle” system. In the catalytic process, electrons transferred from H_2_O_2_ or luminol can be rapidly transferred through a conjugated network of Fe-CN-Fe bonds to accelerate the whole catalytic cycle, and the catalytic function of platinum nanoparticles (Pt NPs) is precisely regulated by environmental pH: the catalytic function of the platinum nanoparticles (Pt NPs) shows a peroxidase-like activity (converting H_2_O_2_ into OH) under acidic conditions and switches to a catalase-like activity (decomposing H_2_O_2_ into H_2_O and O_2_) under neutral alkaline conditions, and the two together form a pH-responsive catalytic system of PB@Pt.and provides a key mechanism basis for designing intelligent catalysis and biomedical application materials.

In addition to this, the design of this work takes into account the possibility of future conversion. Firstly, in terms of stability, PB@Pt-Ab_2_ has an activity retention of >90% after 5 days of storage at 4 °C (see Supplementary Figure S2), demonstrating that it has operational and short-term storage stability for practical applications. Secondly, the synthesis method adopted is simple in process and good in reproducibility, and provides a good foundation for possible process amplification in the future. In particular, the PB@Pt-Ab2 probe demonstrated excellent accuracy and reliability in the detection of VEGF in 5 clinical patient samples (see Supplementary Table S2), verifying the efficacy of the nano-enzyme in a real-world assay scenario.

Overall, this study not only overcomes the incompatibility between HRP and the luminol chemiluminescence reaction conditions but also broadens the application of CAT-like nanozymes in chemiluminescent detection. This work paves the way for the development of high-sensitivity CL systems based on nanozymes for clinical biomarker detection.

## Data Availability

The original contributions presented in the study are included in the article/Supplementary Material, further inquiries can be directed to the corresponding authors.
