# Supplementary material for: Prussian blue-supported platinum nanoparticles as pH-Universal catalase mimics: enabling robust chemiluminescent immunoassay for VEGF in clinical diagnostics
Source: Front Bioeng Biotechnol. 2026 Mar 4;14:1762884. doi: 10.3389/fbioe.2026.1762884 (PMC12996212; doi:10.3389/fbioe.2026.1762884)
Supplement: Supplementary file 1 [file Presentation1.pdf]

Supporting Information

Prussian Blue-Supported Platinum Nanoparticles as pH-Universal Catalase Mimics: Enabling Robust Chemiluminescent Immunoassay for VEGF in Clinical Diagnostics

Ying Yang[a,b,c,d†], *Jiyixuan Li*[a,b,c,d†], *Xiang Hu*[a,b,c†], *Yutao Xiu*[a,b,c], *Miao Zhang*[a,b,c], *Chao Wang*[a,b,c], *Xinlin Liu*[a,b,c], *Bing Liang*[a,b,c], *Dongming Xing*[a,b,c,e]*, *Tingting Zhang*[a,b,c] *

[a] The Affiliated Hospital of Qingdao University, Qingdao University, Qingdao 266071, China

[b] Molecular Medicine Innovation Center, Qingdao University, Qingdao 266071, China

[c] Cancer Institute, Qingdao University, Qingdao 266071, China

[d] School of Basic Medicine, Qingdao University, Qingdao 266071, China

[e] School of Life Sciences, Tsinghua University, Beijing 100084, China

[†] These authors contributed equally to this work.

*Corresponding author

Email: xdm_tsinghua@163.com; zhangtt@qdu.edu.cn

Experimental Section

1. Experimental

1.1. Reagents and materials.

The polyvinylpyrrolidone (PVP, K30) were purchased from Sigma-Aldrich, potassium ferricyanide [$K_3Fe(CN)_6 \cdot 3H_2O$], HCl, H_2O_2 (30%), luminol sodium salt, bovine serum albumin (BSA) and ethanol were purchased from Sinopharm Chemical Reagent Co. Ltd. (Shanghai, China). Horseradish peroxidase (HRP) was bought from Shanghai Sango Biotechnology Co. Vascular endothelial growth factor (VEGF) recombinant antigen, VEGF antibody (Ab_1), VEGF antibody (Ab_2) were products of Jiangsu East-Mab Biomedical technology Co., Ltd. K_2PtCl_6, 5,5dimethyl-1-pyrroline N-oxide (DMPO) and 2,2,6,6-tetramethylpiperidine (TEMP) were obtained from Macklin. All solutions were prepared with ultrapure water. Human serum samples were collected from the Affiliated Hospital of Qingdao University, approved by the Committee on Medical Ethics of the Affiliated Hospital of Qingdao University (Qingdao, China).

1.2. Apparatus.

Scanning electron microscopy (SEM) measurements and energy-dispersive X-ray (EDX) spectrometry were conducted on the JSM-78OOF scanning electron microscope. Transmission electron microscopy (TEM) were taken from a JEM-1200EX microscope. UV–vis absorption spectra were obtained from the Shimadzu UV-1900 UV–vis spectrometer. Powder X-ray diffraction (XRD) analysis was conducted on a Rigaku Smart Lab 3KW. CL measurements were performed with a Chemiluminescent Immunoassay Analyzer (Xiamen Tianzhongda Biotechnology Co., Ltd, TZD-CL-200S).

1.3. EPR Spectroscopy.

The spin trap DMPO (100 mM) dissolved in deionized water was used as the work solution for hydroxyl radical (HO•) detection, and DMPO (100 mM) dissolved in methanol was used as the work solution for superoxide radical ($O_2^{•-}$) detection. In addition, the spin trap TEMP (100 mM) dissolved in deionized water was used as the work solution for singlet molecular oxygen ($^1O_2$) detection. The analysis sample was freshly prepared by mixing PB@Pt (10 μg mL^{-1}) and H_2O_2 (100 mM) in PBS (0.1 M, pH 12.0), followed by addition of different work solutions with equal volume ratio and reacted for 5 min, respectively. Then, the EPR signals were recorded. For the measurement of $O_2^{•-}$, we replaced PBS with methanol.

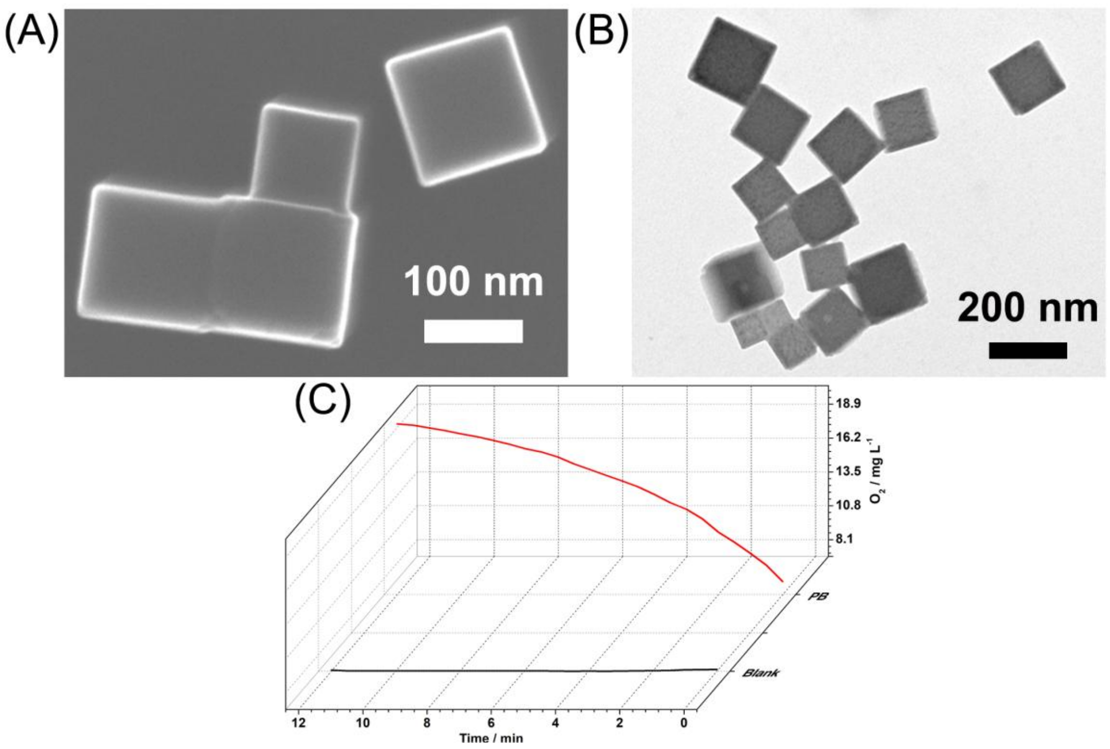

Figure S1. The (A) SEM and (B) TEM images of PB. (C) CAT-mimetic properties of PB in PBS (0.1 M, pH 9.0) containing 50 mM H_2O_2 and 20 μg mL^{-1} PB.

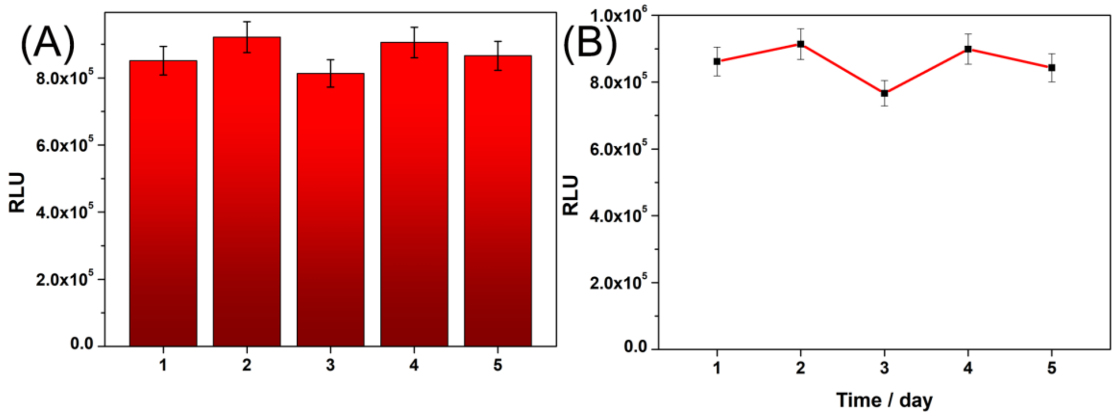

Figure S2. Stability of the proposed CL immunosensor for VEGF detection (A) within one day and (B) over five days.

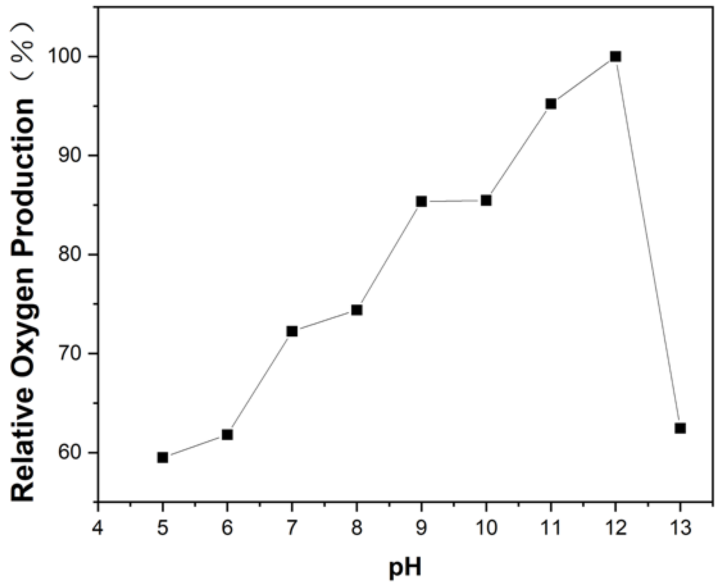

Figure S3. Oxygen production of PB@Pt at the end of the luminol-H_2O_2 catalyzed reaction at different pH (data at pH 12 as a reference, normalized)

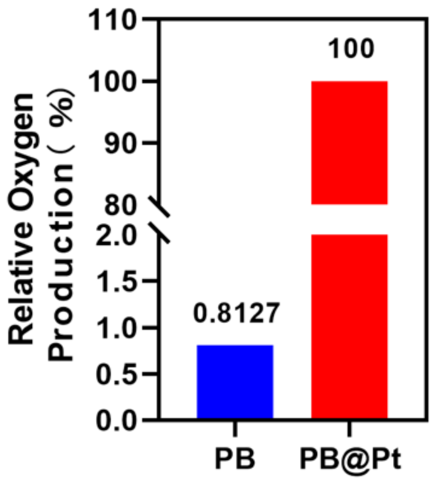

Figure S4. Comparison of relative oxygen production of PB and PB@Pt at the end of the luminol-H_2O_2 catalyzed reaction (pH 12, normalized to PB@Pt data)

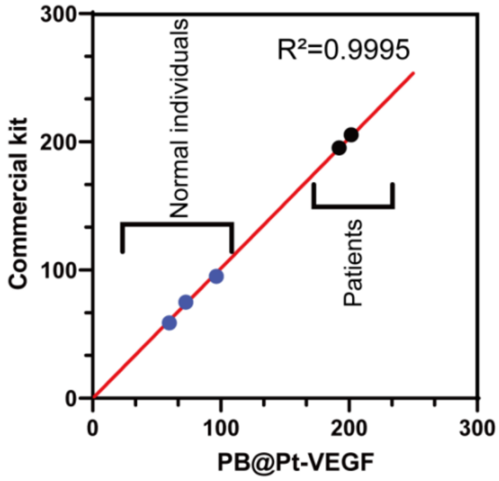

Figure S5. Correlation of VEGF detection results between PB@Pt-VEGF and conventional commercial kits in 5 serum samples (R^2= 0.9995). Each point representations the mean of three replicates.

Table S1. Comparison of the current works for chemiluminescent VEGF determination.

Material	Linear range	Detection limit	Year	Ref
Catalase+CdTe QD/H_2O_2 system	2-35,000 pg mL^{-1}	0.5 pg mL^{-1}	2020	[1]
Apt-VEGF-Apt/ALP	1-20 ng ml^{-1}	1 ng ml^{-1}	2017	[2]

peptide	10-1000 pg mL^{-1}	5.7 pg mL^{-1}	2021	[3]
Mn-PyP	0-15 nM	500 pM	2015	[4]
PB@Pt	5-200 pg mL^{-1}<br>200-2300 pg mL^{-1}	5 pg mL^{-1}		This work

Table S2. Detection of VEGF in real samples.

Sample	Our method[a]	Commercial kit
Serum 1	96.45±2.49	95.17
Serum 2	59.83±1.16	58.82
Serum 3	72.68±1.73	74.91
Serum 4	192.32±4.52	195.35
Serum 5	201.67±3.72	205.39

[a] Mean from three measurements±S.D.

Table S3. Specific information of the human serum samples.

Sample	Age	Diagnosis	Disease Stage
Serum 1	43	Normal	\
Serum 2	46	Normal	\
Serum 3	41	Normal	\
Serum 4	50	Lung Cancer	Stage II
Serum 5	48	Stomach Cancer	Stage II

References

(1) Ghavamipour, F.; Rahmani, H.; Shanehsaz, M.; Khajeh, K.l. Enhanced Sensitivity of Vegf Detection Using Catalase-Mediated Chemiluminescence Immunoassay Based on Cdte Qd/H2o2 System. *Journal of Nanobiotechnology* **2020,** *18*, 1.
(2) Shan, S.; He, Z.; Mao, S.; Jie, M.l. Quantitative Determination of Vegf165 in Cell Culture Medium by Aptamer Sandwich Based Chemiluminescence Assay. *Talanta* **2017,** *171*, 197-203.
(3) Wang, G.; Yin, P.; Wang, J.; Ma, P.l. Specific Heptapeptide Screened from Piii Phage Display Library for Sensitive Enzyme-Linked Chemiluminescence Immunoassay of Vascular Endothelial Growth Factor. *Sensors and Actuators B: Chemical* **2021,** *333*, 129555.
(4) Li, W.; Zhang, Q.; Zhou, H.; Chen, J.l. Chemiluminescence Detection of a Protein through the Aptamer-Controlled Catalysis of a Porphyrin Probe. *Analytical Chemistry* **2015,** *87*, 16, 8336-8341.
