# Supplementary material for: Prussian blue-supported platinum nanoparticles as pH-Universal catalase mimics: enabling robust chemiluminescent immunoassay for VEGF in clinical diagnostics
Source: Front Bioeng Biotechnol. 2026 Mar 4;14:1762884. doi: 10.3389/fbioe.2026.1762884 (PMC12996212; doi:10.3389/fbioe.2026.1762884)
Supplement: Supplementary file 2 [file Table1.doc]

**Table S1.** Comparison of the current works for chemiluminescent VEGF determination.

| Material | Linear range | Detection limit | Year | Ref |
| --- | --- | --- | --- | --- |
| Catalase+CdTe QD/H2O2 system | 2-35,000 pg mL-1 | 0.5 pg mL-1 | 2020 | [49] |
| Apt-VEGF-Apt/ALP | 1-20 ng ml-1 | 1 ng ml -1 | 2017 | [50] |
| peptide | 10-1000 pg mL-1 | 5.7 pg mL-1 | 2021 | [51] |
| Mn-PyP | 0-15 nM | 500 pM | 2015 | [52] |
| PB@Pt | 5-200 pg mL-1  200-2300 pg mL-1 | 5 pg mL-1 |  | This work |

**Table S2.** Detection of VEGF in real samples.

| Sample | Our method^a^ | Commercial kit |
| --- | --- | --- |
| Serum 1 | 96.45±2.49 | 95.17 |
| Serum 2 | 59.83±1.16 | 58.82 |
| Serum 3 | 72.68±1.73 | 74.91 |
| Serum 4 | 192.32±4.52 | 195.35 |
| Serum 5 | 201.67±3.72 | 205.39 |

^a^ Mean from three measurements±S.D.

**Table S3.** Specific information of the human serum samples.

| Sample | Age | Diagnosis | Disease Stage |
| --- | --- | --- | --- |
| Serum 1 | 43 | Normal | \ |
| Serum 2 | 46 | Normal | \ |
| Serum 3 | 41 | Normal | \ |
| Serum 4 | 50 | Lung Cancer | Stage Ⅱ |
| Serum 5 | 48 | Stomach Cancer | Stage Ⅱ |
